# Prevalence and antimicrobial-resistant characterization of *Bacillus cereus* isolated from ready-to-eat rice products in Eastern China

**DOI:** 10.3389/fmicb.2022.964823

**Published:** 2022-07-15

**Authors:** Jiancai Chen, Junyan Zhang, Li Zhan, Honghu Chen, Zhen Zhang, Cheng Huang, Min Yue

**Affiliations:** ^1^Department of Microbiology, Zhejiang Provincial Center for Disease Control and Prevention, Hangzhou, China; ^2^Hainan Institute, Zhejiang University, Hangzhou, China; ^3^Institute of Preventive Veterinary Sciences and Department of Veterinary Medicine, Zhejiang University College of Animal Sciences, Hangzhou, China; ^4^Zhejiang Provincial Key Laboratory of Preventive Veterinary Medicine, Hangzhou, China; ^5^State Key Laboratory for Diagnosis and Treatment of Infectious Diseases, National Clinical Research Center for Infectious Diseases, National Medical Center for Infectious Diseases, The First Affiliated Hospital, College of Medicine, Zhejiang University, Hangzhou, China

**Keywords:** *Bacillus cereus*, quantitative prevalence, rice products, virulence gene, antimicrobial resistance, ERIC-PCR

## Abstract

*Bacillus cereus* is a major food-borne bacterial pathogen in the world, which can cause diarrhea and emetic syndrome. This study aimed to reveal the quantitative prevalence of *B. cereus* in ready-to-eat (RTE) rice products in Eastern China and to gain essential information on the characteristics of *B. cereus* isolates. A total of 91 out of the 1071 samples were positive for *B. cereus*. The contamination level of *B. cereus* in 0.5 % of RTE rice product samples outnumbered 10^3^ CFU/g. The number of *B. cereus* attained 10^5^−10^6^ CFU/g in one sample. The distribution patterns of virulence genes in *B. cereus* isolates were identified. 84.6% of the *B. cereus* isolates had at least one enterotoxin or emetic toxin gene. The predominant pattern was XXV. 9.9% of isolates belonged to it and possessed one enterotoxin gene *entFM*. The occurrence rate of *hblACD* and *nheABC* was 36.3% and 47.3%, respectively. Antimicrobial susceptibility tests revealed a high resistance rate toward penicillin, and 23.1% of the isolates were multi-drug resistant. *B. cereus* isolates were genotyped by using ERIC-PCR. 89 genotypes were determined. The Hunter Gaston Discriminatory Index (HGDI) attained 0.9995. Relationships analysis revealed that Group A *B. cereus* isolates tended to carry *hblA*, *hblC*, *hblD*, *nheA, nheB*, and show resistance to penicillin/trimethoprim/sulfamethoxazole. This study was useful for updating the knowledge of the contamination status of *B. cereus* in RTE rice products in China.

## Introduction

*Bacillus cereus* is a major causative agent of food poisoning outbreaks worldwide. It causes two types of food-borne illnesses, including diarrheal and emetic syndrome. The diarrheal variant is characterized by abdominal pain and watery diarrhea, and is usually linked to the intake of enterotoxin-producing *B. cereus* vegetative cells (e.g., Nhe, Hbl, and CytK). The emetic type, characterized by vomiting symptoms, is caused by ingestion of cereulide, a toxic peptide released by *B. cereus* emetic strains (Senesi and Ghelardi, 2010; Paudyal et al., 2018; Rouzeau-Szynalski et al., 2020; Yue et al., 2020, 2021).

*Bacillus cereus* can persist in a variety of natural environments, including soil and plants, due to its strong survival ability. The endospore resistance to multiple stresses, the formation of biofilms (Glasset et al., 2021; Li et al., 2022a), and even withstanding most cleaning and decontamination processes at food processing steps also help its survival in the environment (Merzougui et al., 2014; Ramarao et al., 2015). *B. cereus* is frequently found in processed products/prepared food items, according to data on the prevalence of *Bacillus* in food and animals in the European Union (European Food Safety Authority, and European Centre for Disease Prevention, and Control., 2017). Rice is a very popular food around the world and is often used as a raw material for the preparation of diet food dishes in many countries. During the cultivation, harvesting, and handling process, rice might be contaminated with vegetative cells and endospores of *B. cereus* (Vasiee et al., 2016; Kindle et al., 2019; Rodrigo et al., 2021). Although vegetative cells can be killed during some cooking processes, such as heating, however, cereulide and endospores generally survive due to high-stress resistance. Under normal conditions, endospores can germinate and become vegetative cells (Rouzeau-Szynalski et al., 2020; Tsugukuni et al., 2020). The possible safety risk of food-borne pathogens in ready-to-eat (RTE) foods is increasingly gaining public attention, because no additional sterilization steps, during cooking, baking, or pasteurization, are normally conducted before the consumption (Chon et al., 2015; Yu et al., 2019; Martelli et al., 2021). Although the contamination status of *B. cereus* in some kinds of food including dairy products, infant foods, aquatic products (Zhang et al., 2017, 2020; Gao et al., 2018; Zhao et al., 2020), etc. has been revealed, the data for *B. cereus* distribution in rice, especially in RTE rice products was still sparse in China. In this study, we investigated the quantitative prevalence of *B. cereus* in RTE rice products sampled in Zhejiang Province, located in Eastern China. The virulence gene distribution profiles, genotyping and antimicrobial susceptibility of these isolates were also studied.

## Materials and methods

### Sampling and isolation of *Bacillus cereus*

A total of 1071 RTE rice product samples were collected from 11 cities covering the whole Zhejiang Province, Eastern China, during 2017−2019. The samples included 756 boiled rice dishes, 91 boiled rice noodles, 105 fried rice noodles, 33 sticky rice rolls, 64 boiled sticky rice dishes, and 22 fried rice cakes. Quantitative detection of *B. cereus* in each sample was performed by using the direct plating method (Liu et al., 2021; Yue et al., 2021; Anwar et al., 2022). Briefly, Twenty-five grams of each sample was suspended in 225 mL of PBS and subsequently homogenized for 2 mins. The homogenate was 10-fold serially diluted in sterilized normal saline. The dilutions were spread on Mannitol-Egg-Yolk-Polymyxin (MYP) agars separately in duplicate. Plates were incubated at 30°C for 24 h. Five presumptive colonies with typical morphology on each plate were selected for further identification. Suspected colonies were then identified using Gram staining and the VITEK2 compact system (BioMerieux, France), followed by rhizoid growth and parasporal crystal formation tests to differentiate *B. cereus* from *Bacillus thuringiensis* and *Bacillus mycoides*. GB/T 4789.14-2014 (Ministry of Health of the People’s Republic of China, 2014) was used to calculate *B. cereus* numbers. One *B. cereus* isolate from each positive sample was stored for further characterization.

### Detection of virulence genes

Genomic DNA was extracted from *B. cereus* by using a bacterial DNA extraction Kit (Omega, United States), according to manufacturer’s instructions. The primers and PCR protocol for eleven virulence genes were used as previously described *ces* (Ehling-Schulz et al., 2005), *hblA* (Zhou et al., 2008), *hblC*, *hblD*, *nheA*, *nheB*, *nheC* (Melnick et al., 2012), *bceT* (in’t Veld et al., 2001), *cytK1*, *cytK2* (Guinebretiere et al., 2006), and *entFM* (Ngamwongsatit et al., 2008). Individual PCR reaction (25 μL) contain 50 ng of DNA template, 0.5 μL of each primer (10 μM), 0.125 U of Taq polymerase (TaKaRa, Japan), 2.5 μL of 10 × PCR buffer (Mg^2+^ free), 1.5 μL of MgCl_2_ (25 mM), and 2 μL of dNTP Mixture (2.5 mM). The amplicon was analyzed with 1% agarose gel. The gels were visualized by a UV Imaging System. A 100 bp DNA ladder (TaKaRa, Japan) was used as a DNA marker.

### Antimicrobial susceptibility tests

Antimicrobial susceptibility assay of *B. cereus* isolates was tested by using the broth micro-dilution minimum inhibitory concentrations (MICs) method according to the standard Clinical and Laboratory Standard Institute (CLSI) guidelines (Clinical and Laboratory Standards Institute, 2015). After 18 h of cultivation on nutrient agar at 37°C, *B. cereus* was suspended in 0.85 per cent (w/v) NaCl solution to 1.0 MCF, followed by dilution with Mueller-Hinton broth to the final concentration of 1∼2 × 10^5^ CFU/mL. Each 100 μL of the bacterial inoculum was added to 96-well plates containing antibiotics and incubated at 37°C for 20 h. Twelve antimicrobials from different classes were employed, including imipenem (1−64 μg/mL), penicillin (0.06−8 μg/mL), chloramphenicol (2−128 μg/mL), ceftriaxone (4−128 μg/mL), vancomycin (1−128 μg/mL), amikacin (8−128 μg/mL), erythrocin (0.25−32 μg/mL), tetracycline (2−32 μg/mL), ciprofloxacin (0.5−16 μg/mL), clindamycin (0.12−16 μg/mL), trimethoprim/sulfamethoxazole (0.5/9.5−16/304 μg/mL), and rifampin (0.5−8 μg/mL). The MIC results were analyzed based on the breakpoints for *Bacillus* species as per CLSI guidelines (Clinical and Laboratory Standards Institute, 2015). The breakpoint for ceftriaxone was from CLSI documents M45-A2 (Clinical and Laboratory Standards Institute, 2010). The isolates resistant to three or more types of antimicrobial classified into different antimicrobial categories were defined as multi-drug resistant (Li et al., 2022b). *Staphylococcus aureus* ATCC 29213 was used as a positive control.

### ERIC-PCR analysis

All 91 *B. cereus* isolates were genotyped by ERIC-PCR using the following primers ERIC-1: 5′-ATGTAAGCTCCTGGGGATTCAC-3′ and ERIC-2: 5′-AAGTAAGTGACTGGGGTGAGCG-3′ (Dorneles et al., 2012; Dorneles et al., 2014). The PCR mixture (25 μL) was 10 mM Tris-HCl (pH 8.3), 50 mM KCl, 4 mM MgCl_2_, 0.3 mM of each dNTP, 1 U of Taq DNA polymerase (Takara, Dalian, China), 0.4 μM of each primer and 75 ng of DNA template. PCR reaction was carried out as follows: 95°C for 3 min, 35 cycles of 94°C for 30 s, 46°C for 40 s, 72°C for 3 min and a final incubation at 72°C for 10 min. Amplicons size was analyzed by 2.0% agarose gel. The gels were visualized by a UV Imaging System. A 100 bp DNA ladder (TaKaRa, Japan) was used as a marker. A 100% of similarity in bands pattern was defined as an ERIC-PCR genotype according to previous report (Magyar et al., 2019).

### Genetic typing analysis

The software BioNumerics 7.6 (Applied Maths, Sint-Martens-Latem, Belgium) was applied to estimate the band size of ERIC-PCR amplicons and analyze the genotypes. Clustering analysis was based on the Dice similarity coefficient and the unweighted pair group method with arithmetic mean (UPGMA). The Hunter and Gaston Diversity Index (HGDI) was calculated to evaluate the discriminatory capability of ERIC-PCR (Shi et al., 2021). Isolates that share 100% similarity of amplicon bands pattern were grouped into one genotype.

### Statistical analysis

Chi-square analysis was performed using the SPSS v 21.0 software package to determine if a significant difference existed in the prevalence distribution of *B. cereus* in different RTE rice products. The *p*-Value of <0.05 was used as a significance level. Relationships between genotype groups and virulence gene distribution, and antibiotic resistance profiles were analyzed by carrying out Pearson’s chi-square test and Fisher’s exact test with the Bonferroni correction.

## Results and discussion

### Quantitative prevalence of *Bacillus cereus* in ready-to-eat rice product

The prevalence of *B. cereus* in 1071 RTE rice product samples examined in this study was described in the Table 1. *B. cereus* was detected in 8.49% (91/1076) of all samples collected, out of which 65/91 (71.4%) were from boiled rice dishes, 6/91 (6.6%) were from boiled rice noodles, 13/91 (14.3%) were from fried rice noodles, 2/91 (2.2%) were from sticky rice roll, 3/91 (3.3%) were from boiled sticky rice dishes and 2/91 (2.2%) were from fried rice cake. According to previously published data, there are significant variances in the detection rate of *B. cereus* in various types of food samples from different regions of the world (Wang et al., 2019; Xu et al., 2020; Qiu et al., 2021; Shi et al., 2021; Wu et al., 2021). The total occurrence rate of *B. cereus* in our study was similar to a previous study in which *B. cereus* was isolated from dairy products, rice and flour products in China (Zhao et al., 2020). The prevalence of *B. cerous* in our study was lower than in a previous study isolated from artisan cheeses made in Mexico and powdered food products in Switzerland (Heini et al., 2018; Adame-Gomez et al., 2020). There was no statistically significant difference (*p* > 0.05) in the prevalence of *B. cereus* across the six types of rice products in our study. *B. cereus* is an opportunistic pathogen found in food. Ingestion of 10^5^−10^8^ vegetative cells or 8 μg of emetic toxin per kg of body weight may lead to gastroenteritis or/and vomiting syndrome in adults (Paananen et al., 2002; Schoeni and Wong, 2005). According to our findings, the number of *B. cereus* detected in 1.0 % fried rice noodles and 0.8% boiled rice meal samples varied from 10^3^ to 10^6^ CFU/g. Food poisoning can occur after consuming a specific amount of these highly contaminated meals (Zeng et al., 2021, 2022).

**TABLE 1:** Quantitative prevalence of *Bacillus cereus* in ready-to-eat rice product.

Samples	No.	Positive	*B. cereus* level (CFU/g)					
			10−10^2^	10^2^−10^3^	10^3^−10^4^	10^4^−10^5^	10^5^−10^6^	ND (<10)
Boiled rice dishes	756	65 (8.6%)	56 (7.4%)	3 (0.4%)	4 (0.5%)	1 (0.1%)	1 (0.1%)	691 (91.4%)
Boiled rice noodles	91	6 (6.6%)	5 (5.5%)	1 (1.1%)	0 (0%)	0 (0%)	0 (0%)	85 (93.4%)
Fried rice noodles	105	13 (12.4%)	9 (8.6%)	3 (2.9%)	1 (1.0%)	0 (0%)	0 (0%)	92 (87.6%)
Sticky rice roll	33	2 (6.1%)	2 (6.1%)	0 (0%)	0 (0%)	0 (0%)	0 (0%)	31 (93.9%)
Boiled sticky rice dishes	64	3 (4.7%)	2 (3.1%)	1 (1.6%)	0 (0%)	0 (0%)	0 (0%)	61 (95.3 %)
Fried rice cake	22	2 (9.1%)	2 (9.1%)	0 (0%)	0 (0%)	0 (0%)	0 (0%)	20 (90.9%)
Total	1071	91 (8.5%)	76 (7.1%)	8 (0.7%)	5 (0.5%)	1 (0.1%)	1 (0.1%)	980 (91.5%)

ND, not detected.

A number of safety criteria for *B. cereus* in RTE meals have been developed. In Canada and the United Kingdom, an acceptable threshold of 10^4^ CFU/g is recommended. In South Korea, Australia, and New Zealand, a lower permissible threshold (10^3^ CFU/g) is adopted (Nsw Food Authority, 2009; Health Canada, 2010; Chon et al., 2015). In our study, 91.5% of the samples had less than 10^3^ CFU/g of *B. cereus*. However, 0.5% of the samples of RTE rice products had more than 10^3^ CFU/g of *B. cereus*, which is more than the acceptable level in some countries. Although *B. cereus* in 91.5% of the samples was <10 CFU/g in our study, *B. cereus* in 0.5 % of RTE rice product samples outnumbered 10^3^ CFU/g that could exceed the acceptable level of some countries.

### Virulence gene profile of *Bacillus cereus* isolates

For many years, scientists have been studying the molecular mechanisms of *B. cereus* virulence. The diarrheal and emetic syndromes have been linked to several virulence factors including, secreted hemolysin BL (Hbl), necrotic enterotoxin (CytK), non-hemolytic enterotoxin (Nhe), enterotoxin FM (EntFM), BceT, and emetic toxin cereulide (Granum and Lund, 1997; Ehling-Schulz et al., 2005; Schoeni and Wong, 2005; Senesi and Ghelardi, 2010). Hbl or Nhe can promote fluid accumulation in ligated rabbit ileal loops due to their hemolytic, dermonecrotic, and vascular permeability activities (Schoeni and Wong, 2005; Griffiths and Schraft, 2017). Both of these enterotoxins comprise the tripartite complex. Three components are required for their maximal biological activity: proteins B, L1 and L2 in Hbl, and proteins A, B, and C in Nhe. Toxin activity has not been detected in any individual components (Arora, 2021; Sornchuer et al., 2022). The genes encoding Hbl and Nhe components are *hblA*, *hblC*, *hblD*, as well as *nheA*, *nheB*, and *nheC*, are located on two different operons (Sastalla et al., 2013). BceT, EntFM, CytK are all single-protein enterotoxins. BceT has cytotoxic, vascular permeability activities and can cause fluid accumulation in ligated mouse ileal loops (Agata et al., 1995). The necrotic enterotoxin CytK, which presents highly cytotoxic, necrotic and hemolytic activities, was initially incriminated in a severe gastroenteritis outbreak causing three patients’ death in France (Lund et al., 2000; Alonzo et al., 2015).

In this study, the distribution of associated encoding genes of the above toxins in *B. cereus* isolates was investigated. 84.6% of the *B. cereus* isolates had at least one enterotoxin or emetic toxin gene. A total of 31 distribution patterns of virulence genes were determined in our study (Table 2). The predominant one was XXV, 9.9% of isolates belonged to it and possessed only one enterotoxin gene *entFM*. The *nheABC* genes were present in 47.3% of the isolates, this frequency was lower than in *B. cereus* isolates from various food source samples and clinical isolates associated with foodborne outbreaks in previous studies (Kim et al., 2011; Glasset et al., 2016; Zhang et al., 2017). The occurrence rate of *hblACD* was 36.3%, which is similar to the previous reports isolated from milk products (Hwang and Park, 2015; Zhang et al., 2017), and it is lower than that of ready-to-eat foods, including vegetables, infant rice flour, rice, and grain-based foods (Chon et al., 2015; Hwang and Park, 2015; Zhang et al., 2017). The coexistence of *hblACD* and *nheABC* was found in 24/91 (26.4%) isolates. Six isolates (6.6%) were found to possess all enterotoxin encoding genes detected in this study.

**TABLE 2:** Virulence genes distribution profile of bacillus cereus isolates.

Patterns	*ces*	*hblA*	*hblC*	*hblD*	*nheA*	*nheB*	*nheC*	*cytK1*	*cytK2*	*bceT*	*entFM*	No. of strains (%)
I	+	**−**	**−**	**−**	**−**	**−**	**−**	**−**	**−**	**−**	**−**	1 (1.1%)
II	+	**−**	**−**	**−**	**−**	**−**	**−**	**−**	**−**	**−**	+	1 (1.1%)
III	+	**−**	**−**	**−**	+	+	+	**−**	+	**−**	**−**	1 (1.1%)
IV	+	**−**	**−**	**−**	+	**−**	+	**−**	**−**	**−**	+	1 (1.1%)
V	**−**	**+**	**+**	**+**	**−**	**−**	**−**	**−**	**−**	**+**	**−**	3 (3.3%)
VI	**−**	**+**	**+**	**−**	**−**	**−**	**−**	**−**	**−**	**−**	+	1 (1.1%)
VII	**−**	**+**	**+**	**+**	**−**	**−**	**−**	**−**	**−**	**+**	**+**	2 (2.2%)
VIII	**−**	**+**	**+**	**+**	**−**	**−**	**−**	**−**	**+**	**−**	**+**	2 (2.2%)
IX	**−**	**+**	**+**	**+**	**−**	**−**	**−**	**−**	**+**	+	+	2 (2.2%)
X	**−**	**−**	**−**	**−**	**+**	**+**	**+**	**−**	**−**	**−**	+	6 (6.6%)
XI	**−**	**−**	**−**	**−**	**+**	**+**	**+**	**−**	**−**	**−**	**−**	3 (3.3%)
XII	**−**	**−**	**−**	**−**	**+**	**+**	**+**	**−**	**+**	**−**	**−**	2 (2.2%)
XIII	**−**	**−**	**−**	**−**	**+**	**+**	**+**	**−**	**−**	+	**−**	3 (3.3%)
XIV	**−**	**−**	**−**	**−**	**+**	**+**	**+**	**−**	**−**	**+**	**+**	1 (1.1%)
XV	**−**	**−**	**−**	**−**	**+**	**+**	**+**	**−**	**+**	**−**	**+**	1 (1.1%)
XVI	**−**	**−**	**−**	**−**	**+**	**+**	**+**	**−**	**+**	**+**	**+**	1 (1.1%)
XVII	**−**	**+**	**−**	**+**	**+**	**+**	**+**	**−**	**−**	**−**	**+**	1 (1.1%)
XVIII	**−**	**+**	**+**	**+**	**+**	**+**	**+**	**−**	**−**	**+**	**+**	7 (7.7%)
XIX	**−**	+	+	+	+	+	+	**−**	**−**	+	**−**	2 (2.2%)
XX	**−**	+	+	+	+	+	+	**−**	**−**	**−**	**−**	3 (3.3%)
XXI	**−**	+	+	+	+	+	+	**−**	+	+	**−**	4 (4.4%)
XXII	**−**	+	+	+	+	+	+	**−**	+	+	+	6 (6.6%)
XXIII	**−**	+	+	+	+	+	+	**−**	+	**−**	**−**	2 (2.2%)
XXIV	**−**	**−**	**−**	**−**	+	**−**	**−**	**−**	+	**−**	**−**	1 (1.1%)
XXV	**−**	**−**	**−**	**−**	**−**	**−**	**−**	**−**	**−**	**−**	+	9 (9.9%)
XXVI	**−**	**−**	**−**	**−**	**−**	**−**	**−**	**−**	+	**−**	**−**	1 (1.1%)
XXVII	**−**	**−**	**−**	**−**	**−**	**−**	**−**	**−**	+	**−**	+	4 (4.4%)
XXVIII	**−**	**−**	+	+	**−**	**−**	**−**	**−**	**−**	**−**	+	1 (1.1%)
XXIX	**−**	**−**	**−**	**−**	**−**	**−**	**−**	**−**	**−**	+	**−**	2 (2.2%)
XXX	**−**	**−**	**−**	**−**	**−**	**−**	**−**	**−**	+	+	+	2 (2.2%)
XXXI	**−**	**−**	**−**	**−**	**−**	**−**	**−**	+	**−**	**−**	+	1 (1.1%)

Two distinct variants of CytK have been reported: CytK1 and CytK2. CytK1 is more harmful than CytK2. Although CytK2 proteins are hemolytic and toxic to Vero cells and human intestinal Caco-2 cells, their toxicity was only around 20% CytK1 (Fagerlund et al., 2004). Furthermore, CytK1 has been linked to major *B. cereus* outbreaks (Fagerlund et al., 2004; Guinebretiere et al., 2006). According to our findings, 33.0% of *B. cereus* isolates had either *cytK1* or *cytK2*. CytK1 was found in one strain, accounting for 3.3% of all *cytK*-positive isolates. A previous study also observed this significant variation in *cytK1* and *cytK2* detection rates in *B. cereus* isolates from Chinese infant meals (Zhang et al., 2017). Foodborne *B. cereus* isolates may be slightly mild when producing diarrhea, according to the researchers. Meanwhile, in China, there was a risk of a *B. cereus* outbreak driven by a *cytK1*-positive strain. The *ces* gene for emetic toxin cereulide production was found in 4.4% of *B. cereus* isolates, consistent with the fact that *B. cereus* with *ces* was rarely isolated from food and environmental materials (Arslan et al., 2014; Chon et al., 2015; Zhang et al., 2017).

### Prevalence of antimicrobial resistance

**FIGURE 1:**
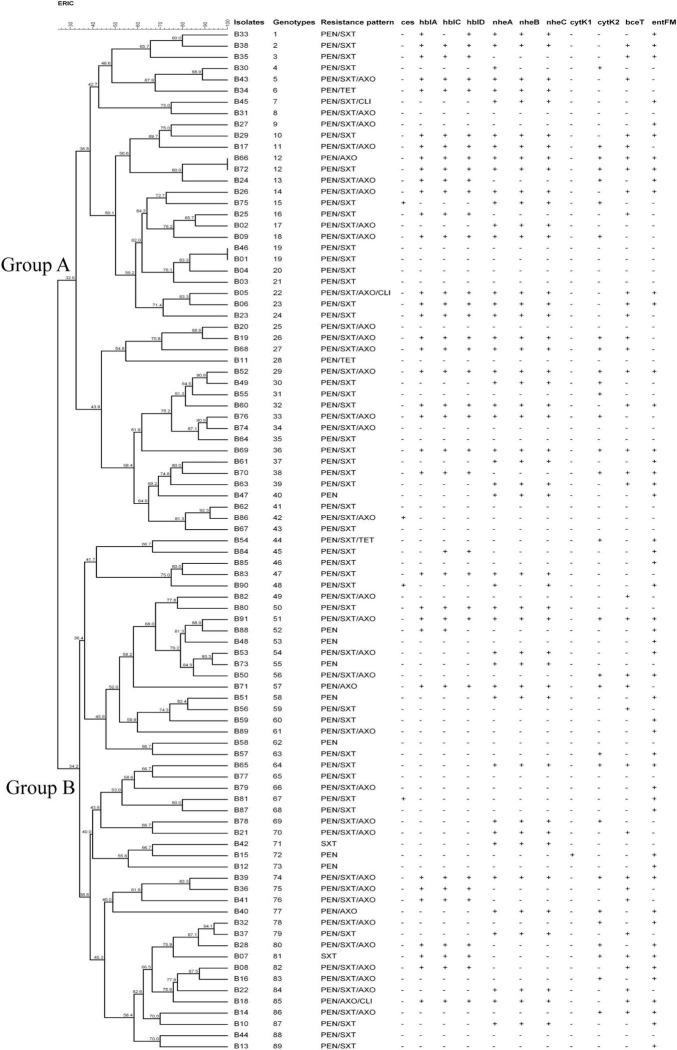
Characteristics of *Bacillus cereus* isolated from ready-to-eat rice products. The UPGMA tree was constructed by using BioNumerics 7.6. PEN, Penicillin; SXT, Trimethoprim/Sulfamethoxazole; AXO, Ceftriaxone; TET, Tetracycline; CLI, Clindamycin.

The antimicrobial susceptibility profile of the *B. cereus* isolates is shown in Table 3. Various susceptibility patterns against 12 types of antibiotics were exhibited. All isolates were susceptible to vancomycin, amikacin, imipenem, and rifampin. 90.1%, 78.0%, 95.6%, and 97.8% of the isolates showed susceptibility to chloramphenicol, erythromycin, tetracycline, and ciprofloxacin, respectively. 97.8% of isolates were resistant to penicillin, consistent with published reports that *B. cereus* isolates from either clinical or food sources were mostly resistant to penicillins (Park et al., 2009; Merzougui et al., 2014; Zhang et al., 2017). A high rate of antimicrobial resistance (84.6% isolates) was also detected against trimethoprim/sulfamethoxazole. All *B. cereus* isolates were classified into eight antibiotic resistance patterns (Figure 1). Resistance to penicillin/trimethoprim/sulfamethoxazole was the most common in our study. 21 isolates (23.1%) were multidrug resistance, with 85.7% were resistant to penicillin/ceftriaxone/trimethoprim/sulfamethoxazole.

**TABLE 3:** Antibiotic susceptibility of *Bacillus cereus* isolates.

Antimicrobial class	Antimicrobial agents	MIC (μg/ml) Interpretive Criteria			No. of isolates (%)		
		Susceptible	Intermediate	Resistant	Susceptible	Intermediate	Resistant
Penicillins	Penicillin	≤0.12	−	≥0.25	2 (2.2)	0 (0)	89 (97.8)
Carbapenems	Imipenem	≤4	8	≥16	91 (100)	0 (0)	0 (0)
Phenicols	Chloramphenicol	≤8	16	≥32	82 (90.1)	9 (9.9)	0 (0)
Cephems	Ceftriaxone	≤8	16−32	≥64	5 (5.5)	49 (53.8)	37 (40.7)
Glycopeptides	Vancomycin	≤4	−	−	91 (100)	0 (0)	0 (0)
Aminoglycosides	Amikacin	≤16	32	≥64	91 (100)	0 (0)	0 (0)
Macrolides	Erythromycin	≤0.5	1−4	≥8	71 (78.0)	20 (22.0)	0 (0)
Tetracyclines	Tetracycline	≤4	8	≥16	87 (95.6)	0 (0)	4 (4.4)
Quinolones	Ciprofloxacin	≤1	2	≥4	89 (97.8)	2 (2.2)	0 (0)
Lincosamides	Clindamycin	≤0.5	1−2	≥4	35 (38.5)	53 (58.2)	3 (3.3)
Folate Pathway Inhibitors	Trimethoprim/Sulfamethoxazole	≤2/38	−	≥4/76	14 (15.4)	0 (0)	77 (84.6)
Ansamycins	Rifampin	≤1	2	≥4	91 (100)	0 (0)	0 (0)

### ERIC-PCR genotyping

All 91 *B. cereus* isolates were fingerprinted and assigned genotypes by using ERIC-PCR. The size of amplicons bands ranged approximately from 100 bp to 2000 bp. Each isolate produces 3-12 DNA bands. Considering 100% similarity in band pattern as a cut-off criteria, 89 genotypes were obtained, as a PCR-mediated fingerprinting typing approach. ERIC-PCR is more straightforward and rapid than PFGE and ribotyping (Dorneles et al., 2014; Magyar et al., 2019). It was initially applied to *B. cereus* for genetic discrimination by PO-REN Hsueh et al. (1999). However, a low number of *B. cereus* strains and low genetic diversity made it insufficient to evaluate the discriminatory capability of ERIC-PCR for *B. cereus*. In subsequent reports, ERIC-PCR was utilized to distinguish the strains of different species in the *Bacillus* genus (Shangkuan et al., 2000). According to our results, the calculated Hunter Gaston Discriminatory Index (HGDI) of ERIC-PCR on *B. cereus* genotyping attained 0.9995 using the optimal PCR system, demonstrating a high discriminatory capability.

Cluster analysis was performed based on UPGMA (Figure 1). Two major genotype groups (Group A and Group B) were defined in our study. 49.5% (45/91) of the isolates belonged to Group A, and 50.5% of the isolates belonged to Group B. Relationships analysis results between genotype groups and virulence gene distribution, antimicrobial resistance profiles demonstrated that there was no association between genotype groups and *nheC* (χ^2^ = 3.167, *p* = 0.075), *bceT* (χ^2^ = 3.167, *p* = 0.075), *cytK1* (χ^2^ = 0.000, *p* = 0.987), *cytK2* (χ^2^ = 0.088, *p* = 0.767), and *ces* (χ^2^ = 0.239, *p* = 1.000). Meanwhile, Group A *B. cereus* tended to carry *hblA* (χ^2^ = 6.018, *p* = 0.014), *hblC* (χ^2^ = 4.09, *p* = 0.043), *hblD* (χ^2^ = 4.967, *p* = 0.026), *nheA* (χ^2^ = 3.963, *p* = 0.046), and *nheB* (χ^2^ = 3.957, *p* = 0.047), and to be resistant to penicillin/trimethoprim/sulfamethoxazole (χ^2^ = 4.643, *p* = 0.031). Considering a limitation of ERIC-PCR as to the repetitive capabilities, genotypic diversity analysis of *B. cereus* based on the more reproducible methods, i.e., MLST and genomic sequencing (Nguyen and Tallent, 2019; Shen et al., 2021; Zhang et al., 2020) might be more informative in the future study.

## Conclusion

Overall, an initial investigation was conducted of the quantitative prevalence and characterization of *B. cereus* isolated from ready-to-eat rice products in Zhejiang Province, Eastern China. A relatively high level of contamination was detected in ready-to-eat rice products, posing a risk of food poisoning and significant public health concern. Differences in the detection rate between the enterotoxin genes and emetic toxin genes revealed that *B. cereus* in ready-to-eat rice products was able to cause diarrhea and lead to food poisoning. *B. cereus* isolates presented high genetic diversity using ERIC-PCR with an HGDI of 0.9995. According to genetic relationships analysis, genotype Group A *B. cereus* isolates tended to carry *hblA*, *hblC*, *hblD*, *nheA, nheB*, and show resistance to penicillin/trimethoprim/sulfamethoxazole. This study provided essential data for addressing the microbial safety of ready-to-eat rice products in China (Peng et al., 2022), accordingly, might improve the appropriate safety criteria and policy.

## Data availability statement

The original contributions presented in this study are included in the article/Supplementary Material, further inquiries can be directed to the corresponding author/s.

## Author contributions

MY did the conceptualization, wrote, reviewed, and edited the manuscript, and carried out the project administration and funding acquisition. JC, JZ, and LZ investigated the data. HC and ZZ validated the data. JC and JZ carried out the data analysis. JZ wrote the original draft preparation. All authors contributed to the article and approved the submitted version.

## Conflict of interest

The authors declare that the research was conducted in the absence of any commercial or financial relationships that could be construed as a potential conflict of interest.

## Publisher’s note

All claims expressed in this article are solely those of the authors and do not necessarily represent those of their affiliated organizations, or those of the publisher, the editors and the reviewers. Any product that may be evaluated in this article, or claim that may be made by its manufacturer, is not guaranteed or endorsed by the publisher.

## Supplementary Material

Supplementary Table 1 The complete meta-data information for isolates in this study.
